# Retraction Note to: Decellularized scaffolds containing hyaluronic acid and EGF for promoting the recovery of skin wounds

**DOI:** 10.1007/s10856-021-06531-9

**Published:** 2021-05-26

**Authors:** Zhengzheng Wu, Yan Tang, Hongdou Fang, Zhongchun Su, Bin Xu, Yongliang Lin, Peng Zhang, Xing Wei

**Affiliations:** 1 Key Lab for Genetic Medicine of Guangdong Province, National Engineering Research Center of Genetic Medicine, Institute of Biomedicine, Jinan University, Guangzhou, 510632, Guangdong China; 2 Multidisciplinary Research Center, College of Science, Shantou University, Shantou, 515063, Guangdong China; 3 Grandhope Biotech Co., Ltd., Building D, #408, Guangzhou International Business Incubator, Guangzhou Science Park, Guangzhou, 510663, Guangdong China

Correction to: **Journal of Materials Science: Materials in Medicine** (2015) 26:59

10.1007/s10856-014-5322-1

The Editor-in-Chief has retracted this article [1] because it contains material that substantially overlaps with two other articles [2, 3].

Author Xing Wei agrees to this retraction but not to the wording of the retraction notice. The editor was not able to obtain a current email address for any of the other authors.

## References

[1] Wu Z, Tang Y, Fang H (2015). Decellularized scaffolds containing hyaluronic acid and EGF for promoting the recovery of skin wounds. J Mater Sci: Mater Med.

[2] Wu Z, Fan L, Xu B, Lin Y, Zhang P, Wei X (2015). Retracted: Use of decellularized scaffolds combined with hyaluronic acid and basic fibroblast growth factor for skin tissue engineering. Tissue Eng Part A.

[3] Su Z, Ma H, Wu Z, Zeng H, Li Z, Wang Y, Liu G, Xu B, Lin Y, Zhang P, Wei X (2014). Enhancement of skin wound healing with decellularized scaffolds loaded with hyaluronic acid and epidermal growth factor. Materials Science and Engineering: C.

